# Serological Survey on the Occurrence of Bacterial Tick-Borne Pathogens in Cattle and Dogs from Bolivian Chaco

**DOI:** 10.3390/vetsci13080838

**Published:** 2026-08-20

**Authors:** Valentina Virginia Ebani, Maribel Mendoza Moreno, Fabrizio Bertelloni, Pablo Abad Rodriguez Farell, Olga Gabriela Palenque Aguilera, Simona Nardoni, Fabio Macchioni

**Affiliations:** 1 Department of Veterinary Science, University of Pisa, Viale delle Piagge 2, 56124 Pisa, Italy; fabrizio.bertelloni@unipi.it (F.B.); simona.nardoni@unipi.it (S.N.); fabio.macchioni@unipi.it (F.M.); 2 Centre for Climate Change Impact, University of Pisa, Via del Borghetto 80, 56124 Pisa, Italy; 3 Facultad Integral del Chaco, Universidad Autónoma Gabriel René Moreno, XF8C+MR5, Av. Humberto Suárez Roca, Camiri 07-03-01, Bolivia; maribelmendoza7moreno@gmail.com (M.M.M.); pablofarell34@gmail.com (P.A.R.F.); palenqueolga9@gmail.com (O.G.P.A.)

**Keywords:** *Anaplasma phagocytophilum*, cattle, *Coxiella burnetii*, dogs, indirect immunofluorescence assay, *Rickettsia rickettsii*, tick-borne pathogens, zoonosis

## Abstract

Bacterial tick-borne pathogens (TBPs) have been reported in Latin America, but information on their prevalence in cattle and dogs in Bolivia are scant. In the present survey, blood serum samples from 95 cattle and 67 dogs living in different communities of Bolivian Chaco, a region in southeastern Bolivia, were tested for antibodies against *Anaplasma phagocytophilum*, *Borrelia burgdorferi* sensu lato (s.l.), *Coxiella burnetii*, *Ehrlichia canis*, and *Rickettsia rickettsii.* Indirect immunofluorescence assays detected 54/95 (56.84%) cattle sera positive to one or more pathogens: 29/95 (30.5%) to *A. phagocytophilum*, 27/95 (28.42%) to *R. rickettsii*, 5/95 (5.26%) to *C. burnetii*; all sera were negative to *B. burgdorferi* s.l. Among canine sera, 11/67 (16.41%) were positive to at least one pathogen: 2/67 (2.98%) to *C. burnetii*, 3/67 (4.48%) to *E. canis*, 6/67 (8.95%) to *R. rickettsii*; all sera were negative to *A. phagocytophilum* and *B. burgdorferi* s.l. To the best of our knowledge, this is the first serological survey investigating exposure to multiple TBPs in cattle and dogs from the Bolivian Chaco. Our findings suggest the circulation of *A. phagocytophilum*, *E. canis*, *C. burnetii,* and Spotted Fever Group *Rickettsia* among the animals living in this area. The results are relevant because these pathogens pose a significant threat to human and animal health and may result in substantial economic losses.

## Simple Summary

Although bacterial tick-borne pathogens have been reported throughout Latin America, information on their prevalence and geographic distribution in cattle and dogs from the Bolivian Chaco, a region in southeastern Bolivia, remains scarce, despite the ecological and veterinary importance of this region. This lack of data represents a significant knowledge gap, as most of these pathogens are relevant to both veterinary and human medicine. They cause substantial economic losses in livestock, and dogs and humans can develop diseases ranging from mild to severe clinical forms, which are often difficult to recognize and diagnose. The present serological survey detected antibodies against bacterial tick-borne pathogens: *Anaplasma phagocytophilum*, *Coxiella burnetii*, *Ehrlichia canis* and *Rickettsia rickettsii* in cattle and dogs from this region. This area is considered higher risk for TBPs due to characteristics such as poverty, inadequate sanitation, and the close coexistence of cattle, dogs, and humans within the same environment, creating favorable conditions for the circulation of tick-borne and other zoonotic pathogens.

## 1. Introduction

Ticks are ectothermic arthropods that rely on ambient temperature to regulate their activity. Climate and weather influence tick survival, seasonal activity, and geographic distribution, with warmer temperatures generally increasing survival and extending the duration of the tick activity season, provided adequate humidity is present [1]. The spread of ticks increases the likelihood of contact between ticks and animals or humans. Consequently, because ticks often act as vectors of pathogens, their expansion is associated with a higher risk and wider distribution of tick-borne diseases (TBDs). Among tick-borne pathogens (TBPs), bacteria of the order *Rickettsiales* are Gram-negative, obligate intracellular organisms that cause severe diseases, mainly in humans. Among them, *Rickettsia rickettsii*, belonging to the rickettsial Spotted Fever Group (SFG), is the etiologic agent of the Rocky Mountain spotted fever, known as Brazilian spotted fever in South America, which affects humans and dogs, transmitted by ticks of the genera *Dermacentor*, *Ixodes*, *Haemaphysalis*, *Amblyomma*, and *Rhipicephalus* [2].

*Anaplasma phagocytophilum* and *Ehrlichia canis* also belong to the order *Rickettsiales*. The first bacterium is mainly transmitted by *Ixodes* ticks worldwide and causes granulocytic anaplasmosis in ruminants, horses, dogs, and humans [3]. The second, *E. canis,* is the primary causative agent of canine monocytic ehrlichiosis (CME), a severe and potentially fatal immunosuppressive disease of dogs and other canids, transmitted globally by the brown dog tick, *Rhipicephalus sanguineus* sensu lato [4]. *Ehrlichia canis* has a worldwide distribution and is endemic in temperate and tropical regions of Europe, Africa, and America. A zoonotic role of *E. canis* has been supposed since it has been detected in Venezuela in human patients with clinical signs resembling those of CME [5].

*Borrelia burgdorferi* sensu lato (s.l.) is a complex of Gram-negative bacteria and belongs to the order *Spirochetales*; it is well known worldwide as the zoonotic agent of Lyme disease. It is transmitted by ticks of the *Ixodes* genus. Small mammals may act as reservoirs, whereas horses, dogs, and humans are accidental hosts that develop clinical signs [6,7].

*Coxiella burnetii*, member of the order *Legionellales*, is the causative agent of Q fever, which induces serious clinical forms in humans and mammals, mainly domestic ruminants, in which reproductive disorders are the main problems. This obligate intracellular Gram-negative bacterium is usually transmitted through inhalation of contaminated aerosol or ingestion of contaminated food [8]; however, it is considered a TBP because ticks of different species are involved in the epidemiology, mainly in wooded or grazing areas [9].

Although the TBPs mentioned above have been reported in Latin America, to our knowledge, information on the prevalence or distribution of bacterial TBPs in cattle and dogs, as well as in humans, in Bolivia is very scarce [10]. This lack of data represents a significant knowledge gap, as these pathogens are relevant to both veterinary and human medicine, and highlights the need for further investigations. These pathogens, in fact, cause substantial economic losses in livestock, and dogs and humans can develop diseases ranging from mild to severe clinical forms, which are often difficult to recognize and diagnose.

Accordingly, the aim of the present survey was to determine the occurrence and prevalence of seropositivity to the tick-borne bacteria *A. phagocytophilum*, *B. burgdorferi* s.l., *C. burnetii*, *E. canis*, and *R. rickettsii* in cattle and/or dogs living in the Bolivian Chaco.

## 2. Materials and Methods

### 2.1. Study Area

The study was conducted in September 2025 in rural communities of the Bolivian Chaco, southeastern Bolivia, located between the latitudes 17°59′–22°21′ south and the longitudes 64°31′–58°51′ west. The region, approximately 127,755 km^2^ in size, is sparsely populated [11] (Gabrielli) and comprises three departments (Chuquisaca, Santa Cruz, and Tarija) and five provinces (Gran Chaco, Luis Calvo, Cordillera, Hernando Siles, and O’Connor).

The Bolivian Chaco is characterized by a semi-arid climate, prolonged dry seasons, seasonal rainfall, and high summer temperatures, frequently exceeding 40 °C [12,13]. The study area is dominated by Chaco dry forests, xerophytic shrublands, and savannas, with seasonal wetlands, small water reservoirs, and artificial ponds representing the main water sources for livestock during the dry season [13]. Livestock production is predominantly based on extensive grazing systems, with cattle freely grazing native vegetation in communal areas throughout the year. Domestic dogs live in close contact with livestock and rural households; they are common in the communities and include both owned free-roaming dogs, which are generally vaccinated against rabies, and unowned stray dogs.

**Figure 1:**
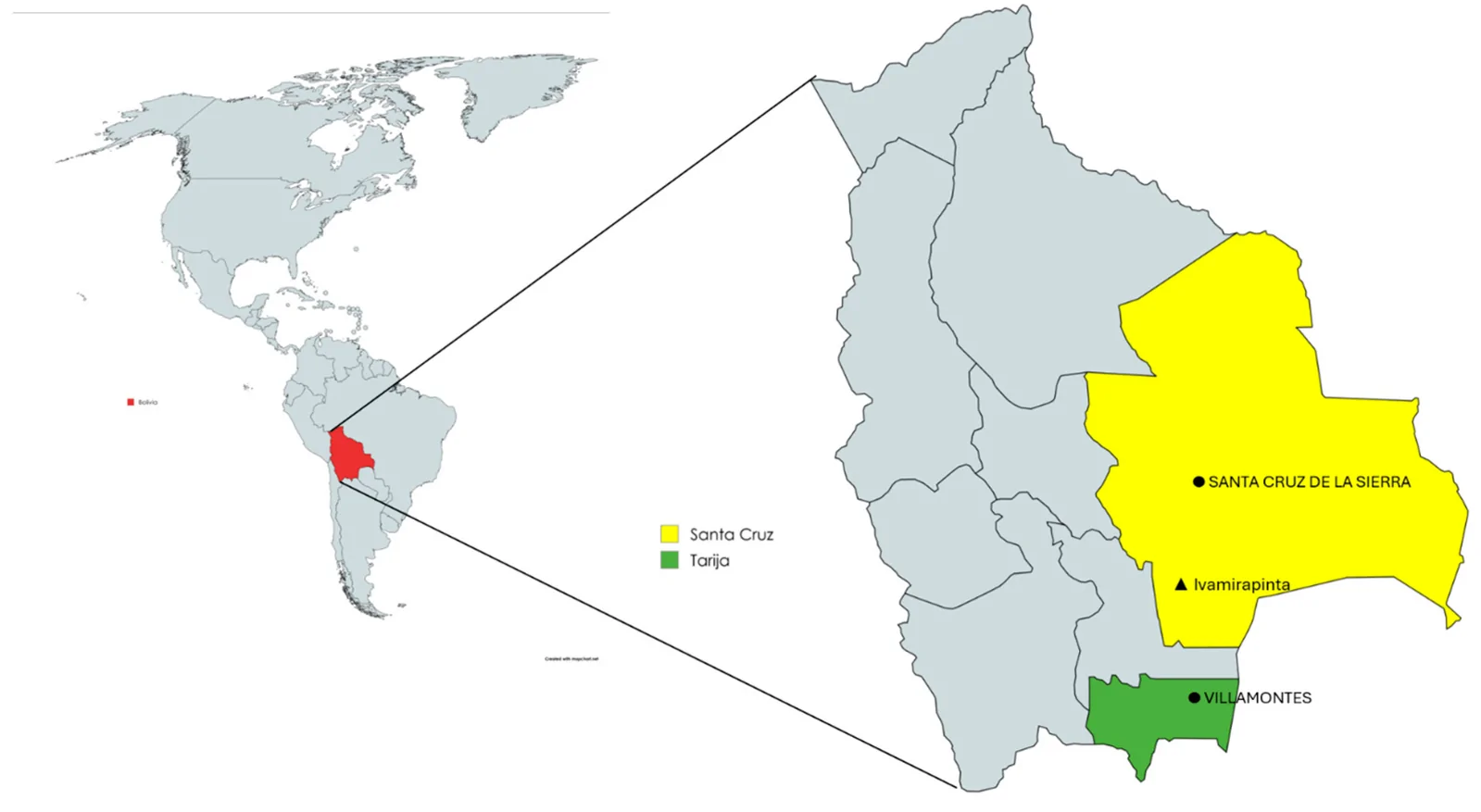
Map of the study area. Red indicates Bolivia, yellow indicates the Department of Santa Cruz with the community of Ivamirapinta, and green indicates the Department of Tarija. The map was generated using MapChart (version 7.11.0).

**Figure 2:**
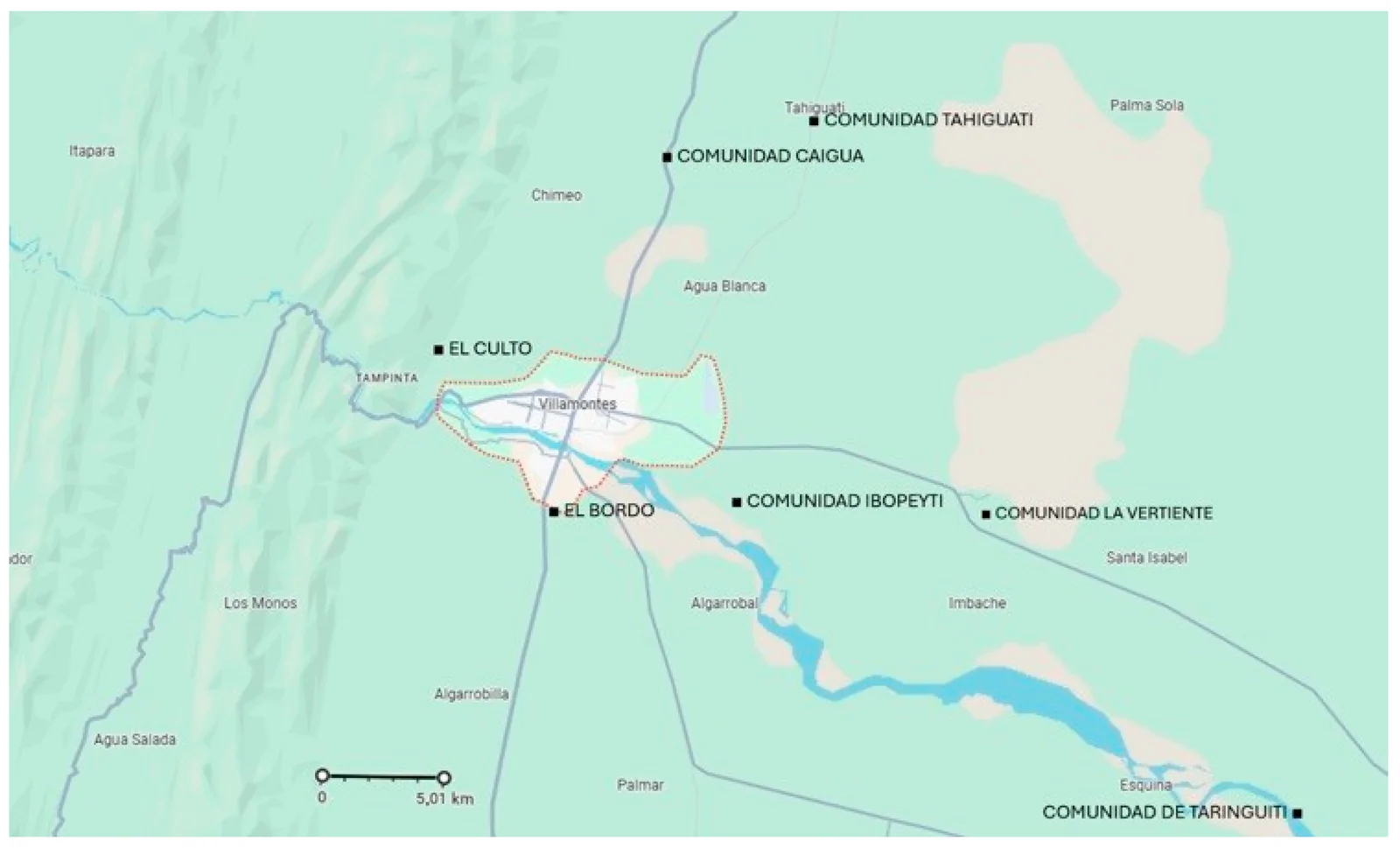
Map of the Department of Tarija showing the communities where sampling was conducted. The map was generated using Google Maps (version 26.33.1.9618914320). (Accessed on 2 July 2026).

Cattle were sampled in the rural communities of Tahiguati, Taringuiti, La Vertiente, Ibopeyti, Caigua, El Bordo, and El Culto, located in the Gran Chaco Province, Tarija Department. Canine sera were collected from dogs in the rural communities of Tahiguati and Ibopeyti (Tarija Department), and Ivamirapinta (Santa Cruz Department) (Figure 1 and Figure 2).

### 2.2. Animal Sampling

A total of 95 cattle and 67 dogs were included in the study. All cattle and dogs included in the study were adults; however, their exact age was not available, as this information was not known by the owners. Blood samples were collected during field surveys conducted in September 2025 from privately owned animals living in rural communities of the Bolivian Chaco. Animals were selected by convenience sampling according to owner availability during field visits.

Blood sampling was performed by trained veterinary personnel of the Facultad Integral del Chaco, Universidad Autónoma Gabriel René Moreno Camiri (UAGRM, Bolivia) within the framework of the academic cooperation agreement between the Universidad Autónoma Gabriel René Moreno and the University of Pisa. Whole blood was collected by jugular venipuncture from cattle and by cephalic venipuncture from dogs using sterile vacuum tubes without anticoagulant. After clot formation, blood samples were centrifuged, sera were separated and stored at −20 °C until serological analysis. During sampling, each serum sample was identified with a unique code and the date of collection, animal species, community of origin and owner identification were recorded on standardized field forms.

### 2.3. Ethical Statement

Blood samples were collected by trained veterinary personnel of the Facultad Integral del Chaco, Universidad Autónoma Gabriel René Moreno (UAGRM), within the framework of the academic cooperation agreement with the University of Pisa. Blood sampling was performed in accordance with the animal health regulations of the Plurinational State of Bolivia and the guidelines established by the Servicio Nacional de Sanidad Agropecuaria e Inocuidad Alimentaria (SENASAG), ensuring compliance with Good Veterinary Practices, biosafety and animal welfare standards. The procedures were certified by the Facultad Integral del Chaco under Certification CITE OF. No. 088/2026 AGP.F.I.CH. Animal owners provided informed consent before sampling.

### 2.4. Serological Tests

Indirect immunofluorescence assay (IFA) was performed to detect antibodies against the pathogens investigated. All bovine and canine sera were diluted 1:40 in phosphate-buffered solution (PBS; pH 7.2) which is the positive cutt-off value. The tests were executed using different commercial IFA slides (Fuller Laboratories Fullerton, Fullerton, CA, USA) coated with the following antigens: *A. phagocytophilum*, *B. burgdorferi* s.l., *C. burnetii*, *E. canis* (used only for canine sera), and *R. rickettsii*, respectively. *C. burnetii* IFA slides included both phase I (left side of each well) and phase II (right side of each well) antigens. Tests were executed following the manufacturers’ instructions and protocols previously described [14].

A fluorescein isothiocyanate-conjugated rabbit anti-bovine IgG (Sigma-Aldrich, Saint Louis, MO, USA) diluted 1:100 in Evans Blue (Sigma-Aldrich, Saint Louis, MO, USA) and a fluorescein isothiocyanate-conjugated rabbit anti-dog IgG (Sigma-Aldrich, Saint Louis, MO, USA) diluted 1:30 in Evans Blue solution were used. Sera, which were positive at 1:40 dilution, were tested at two-fold dilutions to determine the end-point antibody titer.

### 2.5. Statistical Analysis

Prevalence and relative 95% confidence interval (CI) were calculated for each pathogen. Statistical analysis was performed using the χ^2^ test to evaluate the association between serological test results and variables such as communities, targeted pathogen, and animal species. A *p*-value of less than 0.05 was deemed statistically significant. Yates’ continuity correction was applied when necessary to improve the accuracy of the χ^2^ test. Additionally, Cramér’s V was calculated, where appropriate, to measure the strength of the association between categorical variables identified through the χ^2^ analysis.

## 3. Results

### 3.1. Serological Analyses

Among the total 162 sera examined, 65 were positive to at least one pathogen with an overall prevalence of 40.12% (95% CI: 32.57–47.67%).

#### 3.1.1. Cattle

A total of 54/95 (56.84%; 95% CI: 46.68–66.80%) bovine sera were positive for one or more pathogens investigated. In detail, all sera were negative to *B. burgdorferi* s.l., whereas 29/95 (30.5%) were positive to *A. phagocytophilum*, 27/95 (28.42%) to *R. rickettsii*, and 5/95 (5.26%) to *C. burnetii* phase I antigen (Table 1; Table S1). Five/95 (5.26%) sera were positive for two or three pathogens: 3/95 (3.15%) to both *A. phagocytophilum* and *R. rickettsii*, 2/95 (2.17%) to *A. phagocytophilum*, *C. burnetii* and *R. rickettsii*.

No statistically significant differences were observed in the distribution of positive bovine sera across the different communities examined for all pathogens considered (*p* > 0.05). However, it is important to note that the limited number of sera tested and the low number of positive samples from each community may have influenced these results.

#### 3.1.2. Dogs

Among canine sera, 11/67 (16.41%; 95% CI: 7.54–25.28%) were positive for at least one pathogen: 2/67 (2.98%) to *C. burnetii* phase I antigen, 3/67 (4.48%) to *E. canis*, and 6/67 (8.95%) to *R. rickettsii*. All sera were negative to *A. phagocytophilum* and *B. burgdorferi* s.l. (Table 2). No coinfection was detected. All positive dogs were from Ivamirapinta community (Table S2).

The χ^2^ test identified a statistically significant association between animal species and the exposure to certain pathogens. Specifically, antibodies against *A. phagocytophilum* and *R. rickettsii* were detected more frequently in bovines compared to dogs (*p* < 0.01). However, Cramér’s V values for these associations ranged from 0.20 to 0.50, indicating moderate associations. Although the relationships were statistically significant, these findings should be interpreted with caution given the limited sample size, which may affect the precision and generalizability of the estimates.

## 4. Discussion

The results of the present survey are difficult to compare with previous epidemiological data on TBPs in Bolivia, as available information on the occurrence and geographic distribution of these pathogens remains scarce and fragmented. To the best of our knowledge, this study represents the first serological survey investigating exposure to the major tick-borne bacterial pathogens in cattle and dogs from the Bolivian Chaco.

This study was conducted in underserved communities where access to both human and veterinary healthcare is limited. Health surveillance is scarce, and diagnostic facilities and laboratory resources for the detection of infectious diseases are limited or difficult to access. The availability of medicines is also limited, while healthcare and diagnostic facilities are mainly located in urban centers situated at considerable distances from the communities included in the study. Limited economic resources and transportation options, together with the costs associated with travelling to urban centers and obtaining medical or veterinary care, may further restrict access to diagnosis and treatment. Consequently, infectious diseases occurring in these communities may remain undiagnosed or untreated, and their actual circulation and impact may therefore be underestimated. Another important characteristic of the study area is the close coexistence of humans and animals. Companion animals and livestock live near people and frequently share the same spaces and surrounding environment. Such conditions are particularly relevant from a One Health perspective, as humans and animals may be exposed to the same arthropod vectors and zoonotic pathogens.

Our study detected seropositive cattle and dogs for all investigated pathogens, except for *B. burgdorferi* s.l. Lyme disease, caused by this pathogen, is not considered endemic in Latin America and is instead primarily reported in North America and Europe. However, in the early 2000s, sporadic cases of Lyme disease were documented, even if not confirmed, in South American countries, including Argentina, Brazil, Chile, and Mexico [15]. In Bolivia, evidence for Lyme borreliosis is scarce. A seroepidemiological study conducted by Ciceroni et al. (1994) [16] reported a seroprevalence of 10.8% for *B. burgdorferi* s.l. and 24.3% for tick-borne relapsing fever *Borrelia* spp. among residents of the Cordillera Province, representing the first serological evidence of these infections in the country. More recently, Briaçon Ayo (2003) [17] reported confirmed disease human cases in Bolivia. No studies have yet documented *B. burgdorferi* infection in animals in Bolivia, leaving the potential animal reservoirs and transmission cycle in the country largely unknown.

*Coxiella burnetii*-positive samples were found among both cattle and dogs, although with low prevalence rates. The IFA slides employed in this study included both *C. burnetii* phase I and phase II antigens. The seropositive samples exhibited antibodies against phase I antigen, which are typically associated with chronic *C. burnetii* infection [18]. Therefore, the seropositive dogs and cattle were unlikely to have been in the acute phase of infection at the time of sampling These findings provide serological evidence of exposure to *C. burnetii* in the study area. Since *C. burnetii* can be transmitted not only by ticks but also through inhalation or ingestion of contaminated materials, exposure may have occurred through environmental contamination with urine, feces, genital discharges, or milk from infected animals. Although *C. burnetii* has been reported in several South American countries, no data are currently available on its occurrence in Bolivia [19]. The presence of *C. burnetii* infections in humans and animals in countries bordering with Bolivia (Argentina, Brazil, Chile, Paraguay, Peru) [19] supports the biological plausibility of its circulation in Bolivia.

Antibodies reactive to *R. rickettsii* were detected predominantly in cattle (28.42%) and, to a lesser extent, in dogs (8.95%). However, although IFA is considered the gold standard for the serological diagnosis of rickettsioses, it cannot distinguish antibodies against *R. rickettsii* from those directed against other SFG rickettsiae [20]. Therefore, our findings should be interpreted as evidence of exposure to SFG rickettsiae rather than specifically to *R. rickettsii*. *Rickettsia rickettsii* has been identified in Mexico, Panama, Costa Rica, Colombia, Argentina, and Brazil where several cases of Rocky Mountain Spotted Fever were observed in human patients [21,22]. Conversely, data about *R. rickettsii* infections in animals are limited to few studies mainly on canine populations [23,24]. In Bolivia, information on SFG rickettsiae is scarce. Tomassone et al. (2010) [25] reported a *R. rickettsii*-seroprevalence of 68.2% (30/40) among dogs living in the rural, peri-urban, and urban areas of Cochabamba, Bolivia. However molecular analyses performed on blood and ticks collected from the same animals detected *Rickettsia parkeri* and, in one case, *Rickettsia aeschlimannii*. An interesting finding was the higher seroprevalence observed in cattle than in dogs. This difference may be attributable to the geographic distribution of the sampled animals, as nearly all dogs originated from the Ivamirapinta community, where no cattle were sampled. Although dogs in these rural communities are generally free to move, they tend to remain within or close to the settlements and human dwellings. In contrast, cattle are managed under extensive grazing systems and move over larger areas of natural vegetation throughout the year. These different movement patterns may result in exposure to different tick populations or different levels of tick exposure and could partly explain the observed differences in seropositivity. Detailed information on tick infestation and tick species was not available for the animals included in this study; therefore, this hypothesis cannot be confirmed. However, the different prevalence values may reflect that cattle are more frequently exposed to tick species involved in the transmission of *Rickettsia* spp., such as members of the genera *Ixodes*, *Dermacentor*, and *Amblyomma* [26]. Further epidemiological investigations using molecular approaches will be essential to clarify the epidemiology of rickettsioses in the country, particularly given that many SFG *Rickettsia* spp. are recognized human pathogens.

*Anaplasma phagocytophilum*, with 30.52% of positive animals, indicates a high level of exposure to *Anaplasma* spp. in the study population. Several *Anaplasma* species infect cattle, including *A. marginale*, *A. centrale*, *A. bovis*, and *A. phagocytophilum*. Other *Anaplasma* species of unknown pathogenicity, such as *A. platys*-like and *A. capra*, have been detected in cattle, as well [10]. Ogata et al. (2021) [10] found *A. marginale* and *A. platys*-like DNA in pastured cattle in Bolivia, while all tested animals were negative for the other *Anaplasma* species. Our findings may reflect exposure of cattle to *Anaplasma* species other than *A. phagocytophilum*, particularly in samples showing low antibody titers. This interpretation is supported, although not confirmed, by the absence of seroreactivity in the examined dogs. On the other hand, *A. phagocytophilum* has been reported in some countries of Latin America, such as Paraguay [27], Brazil [28], and Panama [28]; therefore, its presence in Bolivia cannot be excluded.

Only 4.48% of the examined dogs had antibodies against *E. canis*. Although a previous survey identified 19/22 (86%) seropositive dogs for this pathogen in Bolivia [29], further investigations are needed to better understand the epidemiology of this TBP in the country. The previous survey reported a higher prevalence; however, its findings are difficult to compare with ours because of the very small number of dogs examined and the lack of reported antibody titer data. *Ehrichia canis* is widely distributed in several South American countries, including Brazil and Venezuela [28], where strains capable of infecting humans have also been reported [5]. In addition, the pathogen has been reported in non-human primates in Brazil [30,31,32]. *Rhipicephalus sanguineus* is the primary vector of *E. canis*; therefore, studies assessing the distribution of this tick species as well as the occurrence of *E. canis* infection in tick populations are essential for clarifying transmission dynamics and evaluating the risk of pathogen spread in Bolivia.

The statistical analysis found significant associations between animal species and the exposure to certain pathogens. Specifically, antibodies against *A. phagocytophilum* and *R. rickettsii* were detected more frequently in bovines compared to dogs (*p* < 0.01). However, these findings should be interpreted with caution given the limited sample size, which may affect the precision and generalizability of the estimates. Moreover, the tested animals were not from the same communities; therefore, the results could be related to the different provenance.

## 5. Conclusions

Indirect immunofluorescence assay is considered the gold standard test for the serological diagnosis of the investigated pathogens, although in some cases positive results could be influenced by cross-reactions with microorganisms that share common antigens. Despite this limitation, our findings provide serological evidence of exposure to *Anaplasma* spp., *E. canis*, SFG *Rickettsia* spp., and *C. burnetii* among cattle and dogs from the study area.

These pathogens represent a significant threat to animal health and can result in substantial economic losses. *Coxiella burnetii* is primarily associated with abortion, stillbirth, and other reproductive disorders, particularly in ruminants. *Anaplasma* spp. and rickettsiae cause diseases in cattle characterized by poor general condition, weight loss, anemia, and reduced milk production, leading to decreased productivity. Collectively, these infections can have a considerable economic impact on the livestock sector through production losses, reproductive failure, increased veterinary costs, and, in some cases, mortality. Given that cattle are a major source of meat and dairy products, these diseases can also affect food security and rural livelihoods.

The same pathogens, together with *E. canis*, can also compromise the health of dogs, although canine infections receive limited attention in Bolivia, where public and veterinary health priorities are largely driven by the country’s socioeconomic challenges. Our findings are important because they provide evidence of the circulation of several zoonotic TBPs in the area investigated. *Coxiella burnetii* and SFG *Rickettsia* spp. are responsible for severe human diseases, underscoring the need for integrated surveillance and control strategies within a One Health framework. Further studies are needed to determine the potential impact of TBPs on cattle health and the Bolivian economy. The study area is characterized by poverty, inadequate sanitation, and close coexistence of cattle, dogs, and humans within the same environment, creating favorable conditions for the circulation of tick-borne and other zoonotic pathogens. Public awareness of the health risks associated with ticks and TBPs is limited. The actual burden of TBP infections in the local human population remains largely unknown. This lack of information, together with the limited diagnostic capacity and epidemiological surveillance in these remote communities, further supports the need for investigations within a One Health framework. Continuous education and awareness programs are essential to improve knowledge of preventive measures and reduce the risk of infection. These initiatives should be regularly delivered by trained professionals, including veterinarians, physicians, biologists, and other public health personnel.

## Figures and Tables

**Table 1:** Number of bovine sera positive for the bacterial pathogens investigated at the given antibody titer.

	Antibody Titers				
	1:40	1:80	1:160	Total	Prevalence (95% CI)
*Anaplasma phagocytophilum*	6	16	7	29	30.52% (21.26–39.78%)
*Borrelia burgdorferi* s.l.	0	0	0	0	0
*Coxiella burnetii*	0	3	2	5	5.26% (0.77–9.75%)
*Rickettsia rickettsii*	11	15	1	27	28.42% (19.35–37.49%)

Legend. 95% CI: 95% confidence interval.

**Table 2:** Number of canine sera positive for the bacterial pathogens investigated at the given antibody titer.

	Antibody Titers				
	1:40	1:80	1:160	Total	Prevalence (95% CI)
*Anaplasma phagocytophilum*	0	0	0	0	0
*Borrelia burgdorferi* s.l.	0	0	0	0	0
*Coxiella burnetii*	2	0	0	2	2.98% (0–7.05%)
*Ehrlichia canis*	1	2	0	3	4.48% (0–9.43%)
*Rickettsia rickettsii*	1	3	2	6	8.95% (2.11–15.79%)

Legend. 95% CI: 95% confidence interval.

## Data Availability

The original contributions presented in this study are included in the article/Supplementary Materials. Further inquiries can be directed to the corresponding author(s).

## Supplementary Materials

The following supporting information can be downloaded at: https://www.mdpi.com/article/10.3390/vetsci13080838/s1, Table S1: Distribution of the sampled cattle and related results in relation to the communities where animals lived.; Table S2: Distribution of the sampled dogs and related results in relation to the communities where animals lived.
