# Supplementary material for: Serological Survey on the Occurrence of Bacterial Tick-Borne Pathogens in Cattle and Dogs from Bolivian Chaco
Source: Vet Sci. 2026 Aug 20;13(8):838. doi: 10.3390/vetsci13080838 (PMC13517835; doi:10.3390/vetsci13080838)
Supplement: Supplementary file 1 [file vetsci-13-00838-s001.zip › vetsci-4489503-supplementary.pdf]

Supplementary material

Serological Survey on the Occurrence of Bacterial Tick-Borne Pathogens in Cattle and Dogs from Bolivian Chaco

Valentina Virginia Ebani [1,2,*], Maribel Mendoza Moreno [3], Fabrizio Bertelloni [1], Pablo Abad Rodriguez Farell [3], Olga Gabriela Palenque Aguilera [3], Simona Nardoni [1], Fabio Macchioni [1]

[1] Department of Veterinary Science, University of Pisa, Viale delle Piagge 2, 56124 Pisa, Italy
[2] Centre for Climate Change Impact, University of Pisa, Via del Borghetto 80, 56124 Pisa, Italy
[3] Facultad Integral del Chaco, Universidad Autónoma Gabriel René Moreno, XF8C+MR5, Av. Humberto Suárez Roca, Camiri, Bolivia

Table S1. Distribution of the sampled cattle and related results in relation to the communities where animals lived.

Community	n. of examined sera	*Anaplasma phagocytophilum*	*Coxiella burnetii*	*Rickettsia rickettsii*	n. coinfection
Taringuiti	10	3 (30.00%; 95% CI: 1.60-58.40%)	0	4 (40%; 95% CI: 9.64-70.36%)	0
El culto	9	1 (11.11%; 95% CI: 0.00-31.64%)	0	3 (33.33%; 95% CI: 2.53-64.13%)	0
Caigua	13	2 (15.38%; 95% CI: 0.00-34.99%)	0	3 (23.07%; 95% CI: 0.17-45.97%)	0
Ibopeyti	13	4 (30.77%; 95% CI: 5.68-55.86%)	0	5 (38.46%; 95% CI: 12.01-64.91%)	1 (7.69%; 95% CI: 0.00-22.17%)
El bordo	9	4 (44.44%; 95% CI: 11.98-76.90%)	0	2 (22.22%; 95% CI: 0.00-49.38%)	0
Tahiguati	22	5 (22.72%; 95% CI: 5.21-40.23%)	1 (4.55%; 95% CI: 0.00-13.26%)	5 (22.72%; 95% CI: 5.21-40.23%)	2 (9.09%; 95% CI: 0.00-21.10%)
La vertiente	19	10 (52.63%; 95% CI: 30.18-75.08%)	4 (21.05%; 95% CI: 2.72-39.38%)	5 (26.31%; 95% CI: 6.51-46.11%)	2 (10.53%; 95% CI: 0.00-24.33%)
**Total**	**95**	**29 (30.53%; 95% CI: 21.27-39.79%)**	**5 (5.26%; 95% CI: 0.77-9.75%)**	**27 (28.42%; 95% CI: 19.35-37.49%)**	**5 (5.26%; 95% CI: 0.77-9.75%)**

Legend. 95% CI: 95% confidence interval

Table S2. Distribution of the sampled dogs and related results in relation to the communities where animals lived.

Community	n. of examined sera	*Coxiella burnetii*	*Ehrlichia canis*	*Rickettsia rickettsii*
Tahiguati	2	0	0	0
Ibopeyti	5	0	0	0
Ivamirapinta	60	2 (3.33%; 95% CI: 0.00-7.87%)	3 (5.00%; 95% CI: 0.00-10.51%)	6 (10.00%; 95% CI: 2.41-17.59%)
**Total**	**67**	**2 (2.98%; 95% CI: 0.00-7.05%)**	**3 (4.48%; 95% CI: 0.00-9.43%)**	**6 (8.96%; 95% CI: 2.12-15.80%)**

Legend. 95% CI: 95% confidence interval
